# Pathophysiology and Treatment Strategies of Acute Myopathy and Muscle Wasting after Sepsis

**DOI:** 10.3390/jcm10091874

**Published:** 2021-04-26

**Authors:** Robert T. Mankowski, Orlando Laitano, Thomas L. Clanton, Scott C. Brakenridge

**Affiliations:** 1 Department of Aging and Geriatric Research, University of Florida, Gainesville, FL 32603, USA; 2 Department of Nutrition and Integrated Physiology, Florida State University, Tallahassee, FL 32306, USA; olaitano@fsu.edu; 3 Department of Applied Physiology and Kinesiology, University of Florida, Gainesville, FL 32611, USA; tclanton@hhp.ufl.edu; 4 Department of Surgery, University of Florida, Gainesville, FL 32610, USA; Scott.Brakenridge@surgery.ufl.edu

**Keywords:** sepsis, myopathy, acute muscle wasting

## Abstract

Sepsis survivors experience a persistent myopathy characterized by skeletal muscle weakness, atrophy, and an inability to repair/regenerate damaged or dysfunctional myofibers. The origins and mechanisms of this persistent sepsis-induced myopathy are likely complex and multifactorial. Nevertheless, the pathobiology is thought to be triggered by the interaction between circulating pathogens and impaired muscle metabolic status. In addition, while in the hospital, septic patients often experience prolonged periods of physical inactivity due to bed rest, which may exacerbate the myopathy. Physical rehabilitation emerges as a potential tool to prevent the decline in physical function in septic patients. Currently, there is no consensus regarding effective rehabilitation strategies for sepsis-induced myopathy. The optimal timing to initiate the rehabilitation intervention currently lacks consensus as well. In this review, we summarize the evidence on the fundamental pathobiological mechanisms of sepsis-induced myopathy and discuss the recent evidence on in-hospital and post-discharge rehabilitation as well as other potential interventions that may prevent physical disability and death of sepsis survivors.

## 1. Introduction

Sepsis is a debilitating disease with medical costs exceeding $17 billion per year and is associated with long-term poor physical outcomes as well as increased all-cause mortality [1] that affects all ages. However, older adults (≥65 years) are affected more commonly, with more frequent long-term complications [1,2]. Higher susceptibility to sepsis in older age may be a consequence of age-related detrimental processes such as immunosenescence and chronic low-grade inflammation. Systemic chronic inflammation, a hallmark of aging, is defined by age-related immune dysregulation due to biological processes such as cellular senescence and oxidative stress without apparent infection [3]. Sustained chronic inflammation, also called “Inflammaging”, is detrimental to health and has been shown to be one of the main contributors to the age-related process of skeletal muscle degeneration (sarcopenia) [3].

One of the main characteristics of sepsis is the acute derangement of pro-inflammatory and anti-inflammatory (immunosuppression) responses in all patient age groups, with older adults not returning to hemostasis after surviving sepsis. The improved implementation of evidence-based intensive care unit (ICU) clinical management has resulted in decreased early hospital mortality. However, many survivors develop chronic critical illness (CCI), with persistent inflammation from which they may never fully recover [2]. Specifically, these patients commonly suffer greater physical complications, necessitating discharge to long-term acute care (LTACs) and/or skilled nursing facilities (SNFs), where they often face recurrent infections requiring re-hospitalization, and prolonged rehabilitation, often suffering an insidious death [2]. A recent report has demonstrated that pro-inflammatory and anti-inflammatory biomarkers are still dysregulated at least 1 year after sepsis [4,5]. This is consistent with our proposed paradigm (based on extensive animal and human studies) that the underlying pathobiology of CCI after sepsis is persistent inflammation, immunosuppression, and catabolism syndrome (PICS) that increases the risk of recurring infections, poor recovery, and death over 1 year after the initial insult [6,7,8,9,10,11].

Sepsis survivors who return home frequently face exacerbation of comorbidities and develop chronic physical disability due to discontinued hospital physical therapy (PT) or occupational therapy (OT) [1]. Persistent inflammation perpetuates physical dysfunction by sustaining ongoing muscle wasting [2,12,13,14], which impairs the performance of daily activities [1,2]. In addition, many of these “sepsis survivors” develop sepsis-induced myopathy, resulting in acute and chronic muscle wasting [15,16] and have dismal long-term outcomes, including muscle atrophy and weakness, severe and persistent disability, an inability to rehabilitate, and a mortality >40% at 1 year [2,12,17,18].

Though the primary biological mechanisms for skeletal muscle abnormalities in this population are still unknown, several candidates have been identified as contributing factors. For example, the negative outcomes in muscle function are presumably exacerbated by high levels of physical inactivity due to bed rest during prolonged ICU admissions. Contributing to this vicious cycle, weak and atrophied muscles become more susceptible to injury [19]. Sepsis has also been shown to disrupt mitochondrial function, responsible for energy production, and damaged mitochondria can be a potent source of damage-associated molecular patterns (DAMPS) contributing to inflammation [13,20]. Additionally, a key element in the self-renewal and plasticity of skeletal muscle to injury and disease is the regenerative capacity of satellite cells (SCs). SCs are unipotent muscle-resident myogenic stem cells [21] localized between the basal lamina and the sarcolemma of myofibers [21,22,23,24]. The health of these cells has been shown to be critically important in the loss of muscle function following sepsis [13]. The relative significance of each of these factors, especially among older adult patients, remains to be elucidated.

Given the biological evidence of sepsis-induced myopathy and failure to recover health status and physical function over at least 1 year in older sepsis survivors, there is an urgent need for practical solutions to improve functional outcomes in this home-bound population at high risk for disability and death. In this review, we will summarize the existing evidence on the fundamental pathobiological mechanisms of sepsis-induced myopathy, recent evidence on in-hospital and post-discharge rehabilitation, and other potential interventions that may prevent physical disability and death of sepsis survivors.

## 2. Pathobiology of Muscle Dysfunction in Sepsis

The most accepted model for the origin of sepsis-induced myopathy is that it is triggered by circulating pathogens and cytokines that signal skeletal muscle pathways associated with halted protein synthesis (due to overproduction of reactive oxygen species) and accelerated protein degradation (due to enhanced proteasome proteolytic degradation and autophagy pathways) [15]. The activation of these pathways leads to decreased muscle mass and likely translates into the loss of force production. However, it is important to highlight that loss of muscle mass (e.g., atrophy) can occur without loss of muscle-specific force. For instance, Lewis and colleagues have shown that when diaphragm muscle mass was reduced by 50% via nutritional deprivation, the specific force remained normal [25]. Nevertheless, if circulating pathogens and cytokines are involved in the myopathy, the ability of skeletal muscles to sense and respond to these factors may be involved in this response.

**Figure 1:**
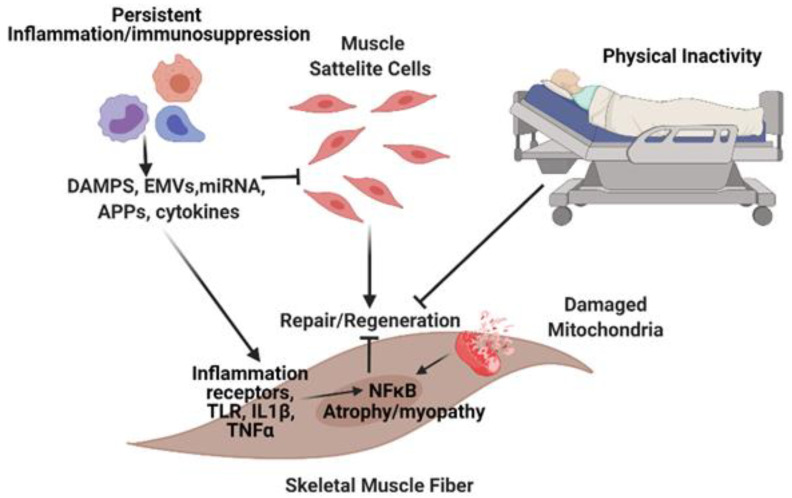
Potential mechanisms triggering sepsis-induced abnormalities in skeletal muscles. Circulating pathogens and cytokines interfere with satellite cell function resulting in impaired muscle-regenerative capacity and atrophy pathways. Damaged mitochondria result in the release of factors that further exacerbates the myopathy. DAMP = damage associated molecular pattern, EMVs = Extracellular Membrane Vesicles; miRNA = microRNA; APP = acute phase proteins.

Muscles sense and respond to circulating pathogens and signals arriving from damaged cells via receptors, particularly toll-like receptors (TLRs) on the sarcolemma. All membrane-bound TLRs, such as TLR4 and interleukin (IL)-1β signal through an adapter protein, myeloid differentiation primary response 88 (MyD88), which eventually activates downstream nuclear factor κB (NF-κB) signaling networks. NFκB is a central player in intracellular inflammatory responses and is a critical link between inflammation and most forms of muscle atrophy and myopathy [26,27]. The expression levels of TLRs are dependent on the type of muscle. For instance, the solei muscles possess more TLR-expressing cells than other limb skeletal muscles, which could reflect the differential susceptibility of different muscle categories to sepsis-induced myopathy [28]. Lipopolysaccharide (LPS) is the most widely described ligand for the TLR family. However, many other pathogens and DAMPS can activate TLRs, including some acute-phase proteins. The importance of TLR-MYD88-NFκB signaling is supported by muscle-wasting models of cancer cachexia [29] and by the demonstration that MyD88 mRNA is inversely correlated to a quadricep’s cross-sectional area and muscle strength during recovery from hip fracture surgery in older adults [30]. Studies have also suggested that MyD88 plays a role in muscle recovery after ischemic injury [31]. In a cohort of older adults, bed rest upregulated TLR4 activity in skeletal muscles, which was associated with outcomes of muscle dysfunction [32]. We have recently shown in rodents that skeletal muscles play an important role in innate immune signaling during sepsis by influencing the concentration of circulatory cytokines and chemokines and by regulating the trafficking of inflammatory cells within the site of infection [33,34]. This indicates that losing skeletal muscles due to myopathy, besides affecting aspects of locomotion, may also interfere with effective immune defense and immune homeostasis (Figure 1).

Another factor that determines the fate of muscle function in sepsis is mitochondrial status. In whole muscle, mitochondrial dysfunction can drive persistent intrinsic tissue inflammation, loss of muscle mass, and loss of force production after sepsis [35]. The breakdown of mitochondria is associated with an elevated release of mitochondrial degradation products and other DAMPs that can interact with receptors to stimulate inflammatory pathways and cytokine secretion in surrounding cells [36]. It is likely that alterations in muscle metabolism that occur during sepsis are involved in other aspects of the myopathy, such as SC dysfunction, which results in an impaired capacity to regenerate and repair the muscle tissue (Figure 1).

SCs are unipotent muscle-resident myogenic stem cells that participate in skeletal muscle regeneration and repair during recovery from injury. They and their immediate surrounding cells and microenvironment are referred to as the SC niche. SCs are characterized by the expression of transcription factor Pax7, which is required for their self-renewal [37,38]. These stem cells are normally in a quiescent (i.e., dormant) state and become activated, generating proliferating MyoD positive progenitors (myoblasts) in response to a variety of environmental stimuli, including pathological conditions that lead to muscle injury. Activated SCs proliferate and differentiate into myoblasts, followed by myocytes. The myocytes can fuse to each other and form new myotubes, which later mature into myofibers. Even though SCs comprise the major tissue-resident stem cell underlying skeletal muscle regeneration, multiple non-satellite myogenic progenitors and non-myogenic populations support the regenerative process by complex interactions within the SC niche [39,40].

Impaired skeletal muscle regrowth and decreased SC content have been demonstrated in human septic patients displaying sustained and progressive atrophy and weakness following hospitalization (Figure 1) [14,41]. These observations are consistent with sepsis-inducing loss of myogenic capacity in SCs from young adult mice. The loss of mitochondrial metabolic homeostasis is also a likely contributor to SC dysfunctions and stability within the muscle. Recent data have demonstrated that SC mitochondrial metabolic state is a regulator of both SC differentiation and muscle inflammation [42]. Whether loss of metabolic control in satellite cells is associated with the loss of SC responsiveness in older septic survivors remains a key knowledge gap.

Despite the metabolic and regenerative barriers in skeletal muscle in response to sepsis, early rehabilitation has been attempted in critically ill patients. We will discuss the main outcomes of these studies in the following section.

## 3. Skeletal Muscle Rehabilitation in Sepsis (in-Hospital and Post-Discharge)

A number of systematic and narrative reviews of randomized clinical trials (RCTs) over the past 8 years have assessed the available published evidence for effective early rehabilitation efforts in reducing the severity of muscle weakness, mobility, and quality of life after critical illness [43,44,45,46,47,48,49,50,51,52,53,54]. Only one systematic review has specifically addressed rehabilitation after sepsis [46]. Whether or not recovery from sepsis and sepsis-induced myopathy is uniquely different from other forms of post-intensive care weakness is not well understood, but there is growing evidence that there are differences in pathophysiology [17,55]. Though all forms of weakness following critical illness present with overlapping clinical features, the severe immune and vascular responses associated with sepsis [12,17] may present a unique underlying pathology requiring specific approaches to prevention and rehabilitation.

In evaluating the results of rehab in all conditions of post-intensive care syndrome, the outcomes of early rehabilitation are mixed but can be roughly divided into three categories: (1) no improvements in long or short-term physical outcomes (RCTs [56,57,58,59,60], reviews: [50,52]); (2) only short-term improvements, e.g., at the time of discharge, (RCTs: [43,61,62], reviews: [45,46,47,48,49,53,54,63]) and (3) long-term improvements (3–6 months), with or without short-term positive outcomes (3–6 months), (RCTs: [64,65], review: [44]).

Overall, the specific rehabilitation protocols used by different investigators have varied widely, making it difficult to compare results among studies. In addition, most investigations have not followed patients beyond discharge. Although the ideal timing to evaluate the success of rehabilitation would presumably be 3 or 6 months, a case can be made that better measures of physical function at discharge are linked to better overall survival and function over subsequent years [52].

Despite all these variations in approach, one variable has emerged that appears to be a major determinant of successful vs. unsuccessful programs. Studies that have started rehabilitation within the first 48–72 h are the only programs that have reported substantially positive physical outcomes, either at discharge or over longer recovery periods [44,47,48,49,66]. This necessity for early intervention is instructive in terms of pathogenesis. It is reasonable to hypothesize that the origins of ICU-acquired weaknesses are, therefore, not simply a long attrition of functioning skeletal muscle over an extended period of time, but rather a response triggered by early events occurring in a very limited time window near the peak of the inflammatory crisis.

To illustrate the wide variety of approaches and the difficulties in assessment, we describe three different rehabilitation protocols with widely varying methodologies and outcomes below. Probably the most famous early rehabilitation attempt that has stimulated many subsequent RCTs was reported in a multicenter trial by Schweickert et al. [61]. Mobilization and rehabilitation began on patients who had been on mechanical ventilation for <72 h. Patients were generally sedated at the time of enrollment. Fourteen percent of the intervention group and sixteen percent of the control group were septic. The intervention consisted of a passive range of motion during sedation and then continued after the interruption of sedation. This was followed by active-assisted and active range-of-motion exercises, beginning in the supine position. Exercise progressed to upright sitting, bed mobility, sitting balance and activities of daily living, sit-to-stand transfer, pre-gait exercise, and eventually walking. Progression was dependent on patient tolerance and stability. A large range of functional activity outcomes, measured at discharge, were positively affected compared to the control group [61].

A second contrasting study was one of the few RCTs performed exclusively on sepsis patients by Kayambu et al. [64]. Patients began rehabilitation within 48 h of sepsis diagnosis, and the therapy included extensive electrical muscle stimulation (EMS), passive range of motion, active range of motion, sitting out of bed, sit-to-stand transfers, and ambulation. The EMS was applied to both the arm and upper and lower leg, including the vastus medialis, vastus lateralis, tibialis anterior, and brachioradialis. The study was double-blinded, and the outcomes measured after 6 months included improvements in self-reported physical function, improved SF-36 scores (a patient-reported health survey), and elevations in IL-10 (evidence for an anti-inflammatory phenotype). Measures of muscle strength were not significantly different. The use of EMS for rehab in critically ill populations has not generally been found to be successful [67,68]. Interestingly, nearly the exact same study was performed in a much smaller group of septic patients [59], but the treatment group showed essentially no differences in outcome at discharge or at 6 months. 

The third study, Eggmann et al. [60], also began therapy within 48 h of critical care admission. None of the patients were identified as septic, and the intervention used, differing from the control group, was 30–60 min of bicycle ergometry per day, starting in the supine position, with some additional low-level resistance exercise. All other aspects of rehabilitation appeared almost identical between control and experimental groups, with approximately the same effort spent in mobilization and movement therapy in both groups. There was no apparent benefit of relatively extensive aerobic bicycle exercise over and above mobilization therapy.

In summary, though there is clearly much more research needed to refine early rehabilitation for sepsis-induced myopathy, there is no magic bullet. The take-home messages are that successful programs, (1) appear to include very early initiation of rehab and (2) careful execution of programmed mobility and range of motion exercises by experienced therapists. In addition, (3) there appears to be no evidence, at this time, that alternative approaches such as extensive resistance exercise, ergometry, or EMS provide a consistent advantage over the careful application of physiotherapy.

## 4. Other Interventions to Prevent Muscle Wasting in Sepsis Survivors

Early rehabilitation is critically important for recovery from a critical illness [61,62]; however, patients who return home could benefit from a structured intervention to keep improving the gains in physical function they obtained during their in-patient stay. Following discharge home, older sepsis survivors demonstrate persisting poor functional status that did not improve over the following year [1,18].

Studies have shown that a 12-week multi-modal home-based intervention including cognitive and physical rehabilitation in middle-aged (41–59 years old) critical illness survivors (25% sepsis patients) was feasible and effective in improving physical and cognitive outcomes [69]. They also found that 92% of patients began an intervention upon discharge from the hospital to their home [69]. Approximately 26% of older patients are discharged directly home from the ICU, and 48% receive home care [10]. In the general SNF rehabilitation population, the large majority of patients achieve clinically meaningful changes in physical performance (87%) and gait speed (78%) [70]; however, patients are at risk of developing problems once discharged home, including loss of function, falls, and hospital readmissions [71,72]. Following in-patient geriatric rehabilitation, patients walk slower and for significantly shorter distances when at home, underscoring the need to help individuals increase the intensity of their activity gained by discharge [73]. While a proportion of patients may receive home-based therapy after discharge from the hospital, these sessions amount to ~1 h/week and often address other domains of disability, such as activities of daily living (ADL) [74]. Sadly, most older patients who go home demonstrate major health impairments, lead sedentary lifestyles, and often live far away from medical and research institutions, and thus are unable to join structured physical activity programs [18]. Currently, no practical approach exists to improve sepsis survivors’ health status and physical function once they return home.

While structured facility-based physical activity is a powerful tool to improve overall health in many health conditions and diseases, most structured physical activity training programs would not be feasible in older sepsis survivors for a few reasons. Firstly, most patients are unlikely to be capable of regularly traveling to participate in structured physical activity programs with multiple visits to intervention sites due to poor health status and distant living locations. Secondly, group-based interventions may be difficult to customize to each individual patient’s level of function. Thirdly, participants may feel less safe in public places, particularly in the era of COVID-19. For these reasons, programs are needed to overcome these limitations and be tailored to individuals in their home environment.

**Figure 2:**
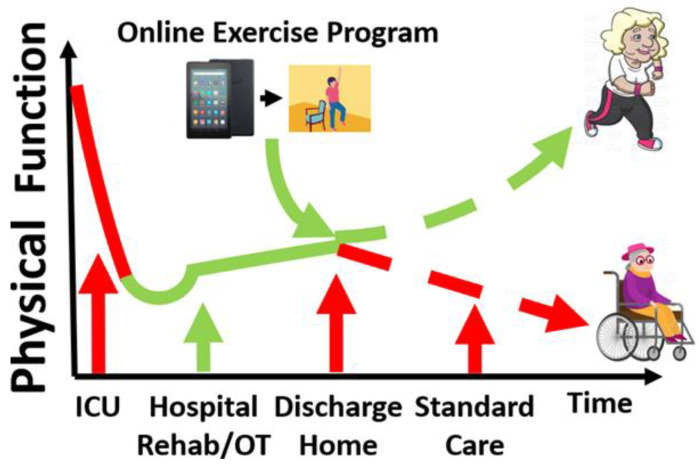
Conceptual illustration of improving physical function in older sepsis survivors. We hypothesize that patients experience early modest physical function improvement with early rehabilitation in the hospital. Upon discharge home, however, physical function plateaus or declines over time (red dashed line) due to limited physical activity. The home-based app-guided exercise training may help the older survivors improve physical function long-term (green dashed line). ICU = intensive care unit, OT = occupational therapy.

In sedentary women (55–70 years old), 12 weeks of home-based physical-activity consulting and monitoring of step-count goals increased their daily step count and decreased sedentary time [75]. Further, 12 weeks of ambulatory sedentary time reduction improved short physical performance battery (SPPB) score and self-reported moderate-to-vigorous physical activity (MVPA) in older men and women [76,77]. Based upon these promising findings, remotely monitored exercise intervention, customized to each participant’s baseline function and rate of progress, may continuously improve physical function in sepsis survivors at home. Thanks to new developments in guided digital health programs, exercise training can be guided by a smartphone ‘app’, and importantly, users can be reminded automatically by the app to perform daily exercises [78,79,80,81]. Figure 2 demonstrates a conceptual model of a remote exercise intervention improving physical function in older sepsis survivors. These novel and practical approaches, which do not require frequent visits to research sites, can be operated and monitored remotely by a research team and could be suitable for older sepsis survivors with their poor physical function and often sizable distance from research facilities. Studies are warranted to test the feasibility and efficacy of app-based and remotely controlled interventions for older sepsis survivors at risk of physical disability and death.

## 5. Conclusions

While sepsis mortality continues to decline through advances in critical care support, long-term outcomes after sepsis remain poor; contributing to these poor post-sepsis outcomes is sepsis-induced myopathy. It is thought that this myopathy is triggered by circulating mediators that signal skeletal muscle pathways associated with decreased protein synthesis and accelerated protein degradation (Figure 1). These pathophysiologic mechanisms lead to muscle mass and force loss. Though promising, results from physical rehabilitation interventions in critical illness remain mixed and need optimization regarding timing, duration, and modalities. Successful interventions will likely require early initiation of assisted mobility, structured range of motion exercises executed by experienced therapists, and remote delivery of these interventions to reach the patients in need.

## Data Availability

Not applicable.
